# Functional characterization of a farnesyl diphosphate synthase from *Dendrobium nobile* Lindl

**DOI:** 10.1186/s13568-022-01470-2

**Published:** 2022-10-06

**Authors:** Daoyong Gong, Bin Wu, Hongting Qin, Dezhao Fu, Shunxing Guo, Bochu Wang, Biao Li

**Affiliations:** 1 Institute of Medicinal Plant Development, Chinese Academy of Medical Sciences and Peking Union Medical College, Beijing, 100193 China; 2 College of Bioengineering of Chongqing University, Chongqing, 400045 People’s Republic of China; 3 Beijing Asia-East Bio-pharmaceutical Co., Ltd, Beijing, 102200 People’s Republic of China

**Keywords:** *Dendrobium nobile* Lindl., Farnesyl diphosphate synthase, Bioinformatics, Methyl jasmonate, Dendrobine

## Abstract

*Dendrobium nobile* Lindl. has been used as a traditional Chinese medicine for a long time, in which the most important compound is dendrobine functioning in a variety of pharmacological activities. Farnesyl diphosphate synthase (FPPS) is one of the key enzymes in the biosynthetic pathway of dendrobine. In this work, we found the expression profiles of *DnFPPS* were correlated with the contents of dendrobine under the methyl jasmonate (MeJA) treatments at different time. Then, the cloning and functional identification of a novel *FPPS* from *D. nobile*. The full length of *DnFPPS* is 1231 bp with an open reading frame of 1047 bp encoding 348 amino acids. The sequence similarity analysis demonstrated that DnFPPS was in the high homology with *Dendrobium huoshanense* and *Dendrobium catenatum* and contained four conserved domains. Phylogenetic analysis showed that DnFPPS was the close to the DhFPPS. Then, DnFPPS was induced to express in *Escherichia coli*, purified, and identified by SDS-PAGE electrophoresis. Gas chromatography-mass spectrometry analysis indicated that DnFPPS could catalyze dimethylallyl pyrophosphate and isopentenyl pyrophosphate to produce farnesyl diphosphate. Taken together, a novel *DnFPPS* was cloned and functionally identified, which supplied a candidate gene for the biosynthetic pathway of dendrobine.

**Supplementary Information:**

The online version contains supplementary material available at 10.1186/s13568-022-01470-2.

## Introduction

*Dendrobium nobile* Lindl. (*D. nobile*) is considered to be a well-known traditional Chinese medicine for hundreds of years (Li et al. 2017), and has been widely used in the therapy of diabetes, gastritis, cancer and age-related diseases (Li et al. 2015; Lv et al. 2020; Nie et al. 2020; Song et al. 2012). The major medicinal components in *D. nobile* are dendrobine, polysaccharides, flavonoids and polyphenols (Bhattacharyya et al. 2015; Li et al. 2021; Luo et al. 2010; Lv et al. 2020; Wang et al. 2019). Among them, as a kind of sesquiterpenoid alkaloid, dendrobine is regarded as the characteristic compound in *D. nobile*, which has important phamacological activities. For instance, dendrobine could alleviate liver injury and attenuate gestational diabetes mellitus in mice (Ci et al. 2020; Feng et al. 2020). Dendrobine has high medical value, however, the key enzyme genes involved in the biosynthesis of dendrobine have rarely been studied in *D. nobile*.

It is well known that sesquiterpenoids are synthesized via the isoprenoid pathway, which is the most abundant and extensive metabolic pathway in all prokaryotes and eukaryotes. A large number of primary and secondary metabolites are produced through this pathway (Wang et al. 2014). Isoprenoids are a very essential and diverse class of molecules that exists in various life forms and is derived from isopentenyl diphosphate (IPP) and its isomer dimethylallyl diphosphate (DMAPP), IPP and DMAPP is a common precursor of the MVA (mevalonate) (Abate et al. 2017) and the MEP (2-*C*-methyl-d-erythritol-4-phosphate) pathways (Lee et al. 2020). In the cytoplasm, the well-characterized MVA pathway synthesizes IPP and DMAPP from acetyl-CoA, which consists of six steps, catalyzed by six corresponding enzymes. Another metabolic pathway for IPP and DMAPP synthesis is the MEP pathway from pyruvate and glyceraldehyde-3-phosphate, which is catalyzed by seven enzymes located in the plastids (Abdul Rahman et al. 2019). The initial reaction of the MVA pathway is catalyzed by acetyl-coenzyme A (CoA) *C*-acetyltransferase (AACT), which is a class II thiolase that condenses two molecules of acetyl-CoA to acetoacetyl-CoA in a reversible reaction (Vögeli et al. 2018). Then, acetoacetyl-CoA is converted to 3-hydroxy-3-methylglutaryl-CoA (HMG-CoA) by HMG synthase (HMGS) (Flynn and Schmidt-Dannert 2018). HMG-CoA is converted to mevalonic acid (MVA) by the enzyme 3-hydroxy-3-methylglutaryl-CoA reductase (HMGR) (Göbel et al. 2019). Subsequently, MVA produces MVA 5-diphosphate in two consecutive reactions catalyzed by MVA kinase (MVK) and phospho-MVA kinase (PMK). The last step of IPP biosynthesis is catalyzed by diphospho-MVA decarboxylase (MPDC), also named MVA-5-diphosphate decarboxylase (PMD). Different isoprenoids share all the early intermediates, up to farnesyl diphosphate through the MVA pathway. The key step of the pathway is the formation of IPP and DMAPP by isopentenyl diphosphate isomerase (IDI). As shown in Additional file 1: Fig. S1, farnesyl diphosphate synthase (FPPS) is a key enzyme in sesquiterpenoids biosynthesis, which catalyzes the consecutive condensations of DMAPP or GPP with IPP to produce FPP (Srivastava et al. 2015; Wang et al. 2014). FPP is located at the first multi-branch point of the various terpenoid biosynthetic pathways such as sesquiterpenes, triterpenes, carotenoids, gibberellins, sterols, dolichols and ubiquinone (Dhar et al. 2013; Wei et al. 2020).

To date, many *FPPSs* have been cloned and characterized in human, some higher plants, yeast and fungi (Kim et al. 2018; Lee et al. 2017a; Rubat et al. 2017; Sharifirad et al. 2016). Studies indicated that the nucleotide sequence length of *FPPS* varies from 1.0 to 2.1 kb. FPPS have different isoforms in some orgnisms and play important functions. For example, in human, only one *FPPS* was encoded containing two isoforms (Romanelli et al. 2009). In *Arabidopsis thaliana*, there are two *fpps* genes containing three isoforms, and knockout of two genes at the same time would lead to the death of plant (Closa et al. 2010; Keim et al. 2012). Moreover, there was a study reported that the mycorrhizal fungus MF23 (*Mycena* sp.) helped to increase the content of dendrobine in *D. nobile*, while the expression of a *FPPS* was upregulated, indicating the potential role of the *FPPS* in the dendrobine biosynthesis (Li et al. 2017). However, there is no report about the cloning and functional identification of *DnFPPS*. In this study, we further analyzed the expression profiles of *DnFPPS* in different and under methyl jasmonate treatments. In addition, the correlation between the expression profiles of *DnFPPS*, and contents of dendrobine under methyl jasmonate (MeJA) treatments in *D. nobile* were also investigated. Then, *DnFPPS* was cloned by rapid-amplification of cDNA ends (RACE) approach, analyzed the sequence characterization by bioinformatics approach, and detected the enzyme activity by Chromatography-Mass Spectrometer (GC-MS). This study would be helpful for the biosynthesis of dendrobine through enginering in the future work.

## Materials and methods

### Materials and MeJA treatment

*Dendrobium nobile* Lindl. seedings were obtained from Chishui Xintian humantang Pharmaceutical Company Limited (Guizhou, China). Then, seedlings were transplanted into plastic pots (20 cm height and 20 cm diameter) containing a 2:1 mixture of pine bark and sawdust. The plant materials were grown in a glass house at 24–28 °C under natural light and watered twice a week. Subsequently, the biennial plant materials (average height, 25 ± 10 cm, n = 9; number of mature green leaves, 7 ± 3 cm, n = 9) were treated with MeJA (Sigma-Aldrich), and each group consisted of at least five plants. Plant materials were sprayed thoroughly until run-off with 100 µM of MeJA solution prepared in MilliQ water containing 0.5% (v/v) absolute ethalnol and covered with polyethylene bags. After 0.5 h of treatment, the polyethylene bags were taken out and then the plant materials were transferred to the glass house. The control plant materials were sprayed with same solution without MeJA, and the other steps were the same as the MeJA spraying group, and isolated from the MeJA-treated plant materials, but the plant materials under the same treatment and growth conditions as the MeJA-treated plant materials. After treatment at the same time, the stems and leaves of the plant materials were harvested, immediately frozen them in liquid nitrogen, and stored at -80 °C ultra-low temperature refrigerator to further separate metabolites and RNA. The leaves were collected at 0 h, 0.5 h, 1 h, 2 h, 4 h, 8 h, 16 h, 24 h, 7 d, 14 d, 21 d and 28 d after incubation for analysis of expression level of *DnFPPS*. The stems were collected at 0 d, 7 d, 14 d, 21 d and 28 d after incubation for determination the content of dendrobine. In addition, *DnFPPS* expression profile was detected by collecting materials from different tissues (roots, stems, leaves and flowers) of the uninduced group.

### RNA extraction, detection and reverse transcription

Total RNA of *D. nobile* samples, including different tissue and MeJA-treated were extracted according to the RNAprep Pure Plant Kit (Polysaccharides and Polyphenolics-rich) (TIANGEN, China) manufacturer’s instructions. To generate the cDNA representative of MeJA-induced transcription, *D. nobile* plants treated with 100 µM MeJA were used as the test samples, whereas non-treated plants were used as the control samples. Then, leaf samples from the two groups of plants (the MeJA-treated plants and the non MeJA-treated plants) were collected for RNA extraction. After extration, total RNA was detected by electrophoresis on a 1.0% agarose gel. Then, the concentration and quality of total RNA were determined the Thermo Scientific NanoDrop 2000/2000c Spectrophotometer. Finally, reverse transcription was performed according to the Thermo Scientific RevertAid First Strand cDNA Synthesis Kit (Thermo Fisher, USA) following the manufacturer’s instructions.

### Analysis of the expression profiles of *DnFPPS* by quantitative reverse transcription PCR (qRT-PCR)

Firsly, reverse transcription was performed using 500 ng total RNA for each sample and the primers used were presented in the Table S1 (FPPS-1-RT-F/R). The resulting cDNA was diluted 10-fold. qRT-PCR reactions (15 µL volume) consisted of 7.5 µL TB Green^®^ Premix Ex Taq™ II (Tli RNaseH Plus), 2 µL of diluted cDNA, 10 mM of each forward and reverse gene-specific primer, and sterile ddH_2_O. Then, qRT-PCR was performed by LightCycler^®^ 480 II (Roche) using the conditions: 95 °C for 5 min, 40 cycles of 95 °C for 30 s, 60 °C for 30 s and 72 °C for 15 s.

The GAPDH was used as the endogenous control for qRT-PCR because it is relatively stable in samples of different tissues and MeJA treatment. Subsequently, the melting curves were analyzed at the dissociation step to test the specificity of amplification. Relative gene expression was analyzed following the 2^−ΔΔCt^ method (Kenneth and Thomas 2002) .

### Extraction and analysis of dendrobine from *D. nobile* under MeJA treatments

The stems collected after incubation for 0 d, 7 d, 14 d, 21 d and 28 d were collected for determination of dendrobine content. After the stems of each treatment were dried at 55 °C, they were thoroughly mixed with a pestle and mortar and ground into a fine powder. The dendrobine standard and internal standard naphthalene used in the experiment were purchased from Sinopharm Chemical Reagent Co., Ltd. and China Food and Drug Control Institute respectively. The GC analysis samples were prepared in accordance with the Chinese Pharmacopoeia (2020). Chromatography was performed on an Agilent 6890 GC-FID, using an Agilent DB-1 capillary column (0.25 μm × 0.25 mm × 30 m) and nitrogen as the carrier gas.

The experimental sample was repeated at least three times. The samples were analyzed in random order. Each injection of 1 µL for analysis. The parameters and methods of gas chromatography analysis refer to the Chinese Pharmacopoeia (2020). The flame ionization detection was used to extract the components of the total ion chromatogram. The relative correction factor of naphthalene and dendrobine was obtained (f = 0.002734). The linear regression equation y = 0.1176x + 0.0907 (R^2^ = 0.9991) proved that the concentration of dendrobine was linear, and the peak area is in the range of 4.6−23.0 mg·L^−1^.

### Cloning of a *DnFPPS* from *D. nobile*

The *FPPS* was obtained from our transcriptome data as described previously (Li et al. 2017). Because the sequences did not contain intact ORF, we amplified the 3’ and 5’ sequences of gene by RACE approach. The RACE primers were subsequently designed using Primer Premier 5.0 (Additional file 1: Table S1). After PCR amplification, the PCR product was purified and cloned into pMD19-T vectors (TAKARA, Japan), and sequenced by Sanger method (GENEWIZ). After aligning and assembly, the full-length cDNA sequences of the *DnFPPS* was deduced. Then, the full-length of *DnFPPS* was amplified by PCR using a pair of specific primers containing restriction sites (Additional file 1: Table S1). Finally, the PCR product was purified and cloned into pMD19-T vectors (TAKARA, Japan), and sequenced by Sanger method (GENEWIZ).

### Bioinformatics analysis

The cloned sequences were compared and analyzed via online BLASTn. The nucleotide sequence, deduced amino acid sequence and ORF were analyzed by DNAstar and ORF Finder, and the sequence comparison was conducted through a database search using BLASTp. DnFPPS and other FPPSs retrieved from NCBI were aligned using Clustal W. A phylogenetic tree was constructed using maximum likelihood method by MEGA 7 (Additional file 1: Table S2) (Han et al. 2021). The theoretical isoelectric point and molecular weight of the protein were predicted by the ExPASy (Bhattacharya et al. 2018). Multiple sequence alignments were performed using DNAman Version 6 (Lynnon Biosoft, San Ramon, CA, USA) (Yang et al. 2020), and spatial structural modeling was accomplished using ExPASy’s Swiss-Model and PyMOL software (Yu et al. 2021).

### Construction of protein expression vector and transformation into ***Escherichia coli*** strains

The specific primers containing the restriction sites were designed with Primer Premier 5.0 (Additional file 1: Table S1), and the double restriction sites were introduced by PCR amplification. PCR was performed in 50 mL volume, containing 1× PCR buffer, 0.4 mM of the two primers (FPPS-1-F-BamHI/ FPPS-1-R-xhoI), 0.4 mM dNTP, 2.5 units of LA-Taq DNA polymerase (TAKARA), and 1 mL the *D. nobile* cDNA under the condition: 95 ℃ for 3 min, 35 cycles of 95 ℃ for 30 s, 67 ℃ for 1 min, and 72 ℃ for 2 min. Then, the PCR products and the expression vector pET-28a were double digested with BamHI/XhoI, and the digested products were used for the ligation reaction after gel recovery to form pET-28a-DnFPPS. Finally, the protein expression vector pET-28a-DnFPPS was transformed into *E. coli* BL21(DE3) competent cells by heat shock approach, and screened by Luria-Bertani (LB) plates with 50 µg/mL kanamycin (Gao et al. 2019) The transformed colonies were confirmed by PCR and sequencing, to confirm the transformation of pET-28a-DnFPPS.

### Recombinant FPPS protein induction and purification

The transformed colonies were cultured in 150 mL of LB medium and incubated at 37 °C (150–200 rpm) until the culture reached OD_600_ = 0.8−1.0. Then 0.5 mM IPTG was added and the induction temperature was set at 16 °C overnight. Then, the bacteria were harvested by centrifugation at 4000×*g* for 30 min. Subsequently, 0.02 M PBS (phosphate buffer saline) was added, and the cells were broken by an ultrasonic cell disruptor. Finally, the his-tagged protein purification kit (Cwbio, China) was used to purify the crude proteins. SDS-PAGE was used to detect the expression of the recombinant protein (Zha et al. 2016). In brief, SDS-PAGE was performed at a 10% electrophoresis gel and stained with Coomassie brilliant blue. After bleaching, a ChemiDoc^™^ imaging system (BIO-RAD) was used to observe the results.

### Enzyme activity detection and product identification by GC−MS

The DnFPPS activity was determined by transforming IPP and DMAPP to produce FPP. We put 2 mM MgCl_2_, 5 mM of DL-Dithiothreitol, 50 µM of DMAPP, 50 µM of IPP, 50 mM of MOPS (pH 7.5) and an appropriate amount of the DnFPPS (0.483 mg/mL) into the reaction mixture with a total volume of 200 µL. The product of the transformed colonies harboring pET-28a was used as a negative control. After incubating overnight at 37 °C, the mixture was extracted with l-butanol saturated with water, and the diphosphate group was hydrolyzed with potato acid phosphatase at 37 °C.The hydrolyzed products were extracted with n-hexane, filtered with a 0.22 μm membrane filter, loaded on an Agilent J&W HP-5MS column, eluted with N_2_ at 1.2 mL/min, detected by Gas Chromatography (GC) (Models 7890B; Agilent Technologies, Palo Alto, CA, USA). The temperature of the oven was first kept at 80 °C for 1 min, then increased to 220 °C at a rate of 10 °C/min, and finally held at this temperature for 10 min. The injector and transmission line temperatures were set at 200 and 250 °C, respectively. The detection of the analytes was performed using a mass spectrometer (MS) (Models 5977 A; Agilent Technologies) and the Nist11 database was used to analyze the compounds.

### Statistical analysis

SPSS 19.0 (Chicago, IL, USA) software was used to analyze the significant differences, and the correlation between gene expression profiles and the contents of dendrobine in samples of different time points at the 5% level using unpaired t-test (two-tailed).

## Results

### Analysis of expression level of *DnFPPS* and content of dendrobine under MeJA treatments

**Fig. 1:**
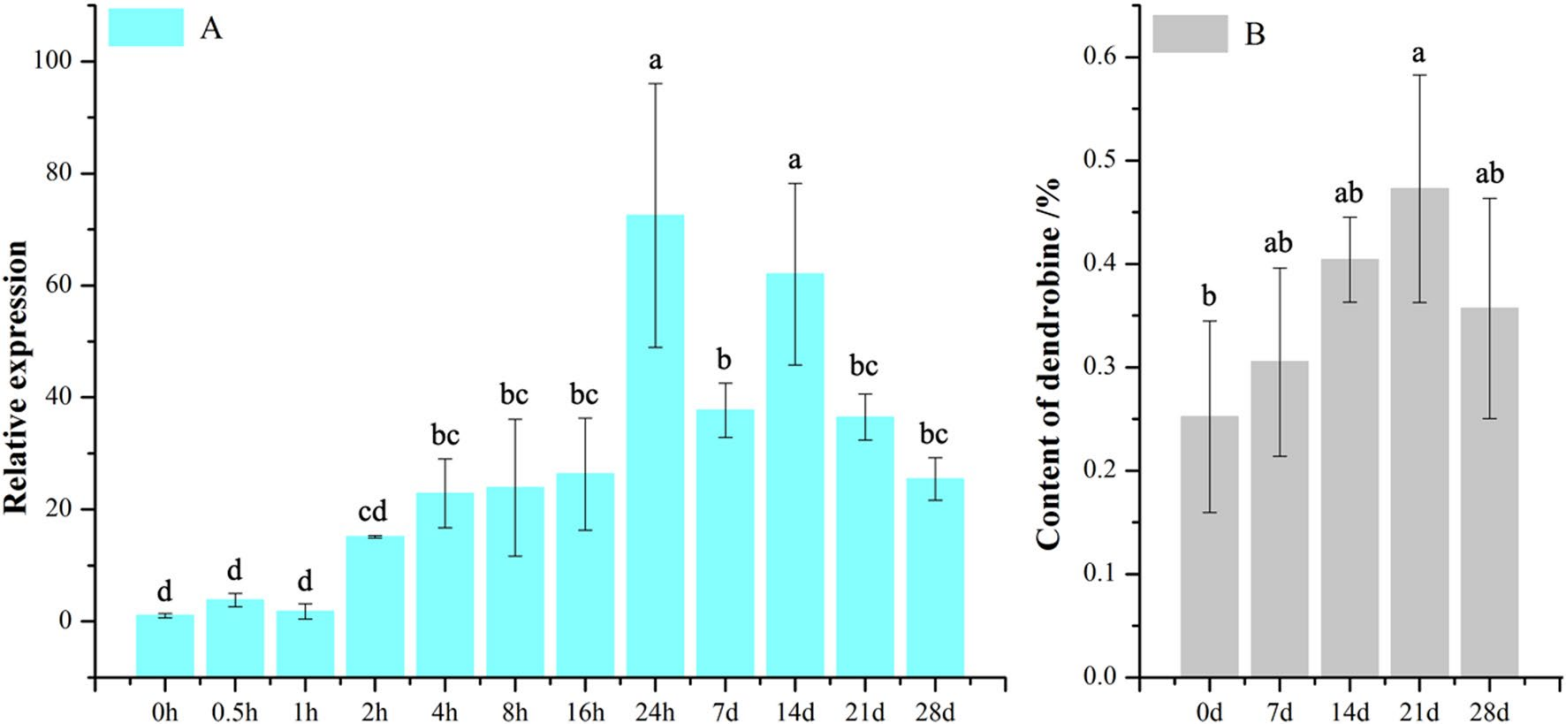
The relative expression of *DnFPPS* at different time points (cyan column) and the content of dendrobine (gray column) in *D.nobile* after MeJA treatment

qRT-PCR was used to detect the transcription level of *DnFPPS* at different time after MeJA treatment. As shown in Fig. 1 (cyan column), the expression level of *DnFPPS* from 0 to 1 h was relatively low. Then, the expression level of *DnFPPS* was increased after 2 h, and reached the highest at 14 d. The effect of MeJA induction on the content of dendrobine in *D. nobile* is shown in Fig. 1. There was a significant difference in the content of dendrobine in the control group and the experimental group, indicating that MeJA can effectively stimulate the biosynthesis of dendrobine (Fig. 1 (gray column)). Moreover, the expression level of *DnFPPS* was positively correlated with the amount of dendrobine. Therefore, we speculated that *DnFPPS* might participat in dendrobine biosynthesis through MeJA-mediated signal pathway.

The expression profiles of *DnFPPS* in different tissues of *D. nobile* showed the stem > leaf > root > flower (Additional file 1: Fig. S2), consistent the amount of dendrobine in different tissue (Li et al. 2017), which also implied *DnFPPS* was related to the biosynthetic of dendrobine. Therefore, we further cloned and validated the function of *DnFPPS*.

### Cloning of full-length cDNA of *DnFPPS*

Through our previous transcriptome data, the putative 615 bp fragment of *DnFPPS* was obtained. In order to obtain the full-length sequence of *DnFPPS*, the SMARTer^®^ RACE 5’/3’ kit was used to perform PCR amplification according to the instructions. *DnFPPS* was sequenced after purification. Sequence analysis showed that the length of the cloned DNA was 1231 bp, including the ORF flanked by 80-bp 5’-UTR and 104-bp 3’-UTR. ORF Finder showed that *DnFPPS* contained an ORF with a length of 1047 bp (Electrophoresis results are shown in Additional file 1: Fig. S3) encoding a protein with 348 amino acids. The calculated molecular weight of *DnFPPS* was about 40.31 kDa, and the theoretical isoelectric point was 5.09. The nucleotide sequence of *DnFPPS* has been submitted to the GenBank database under the accession number MZ044976.

### Sequence analysis and homology model of the DnFPPS protein

**Fig. 2:**
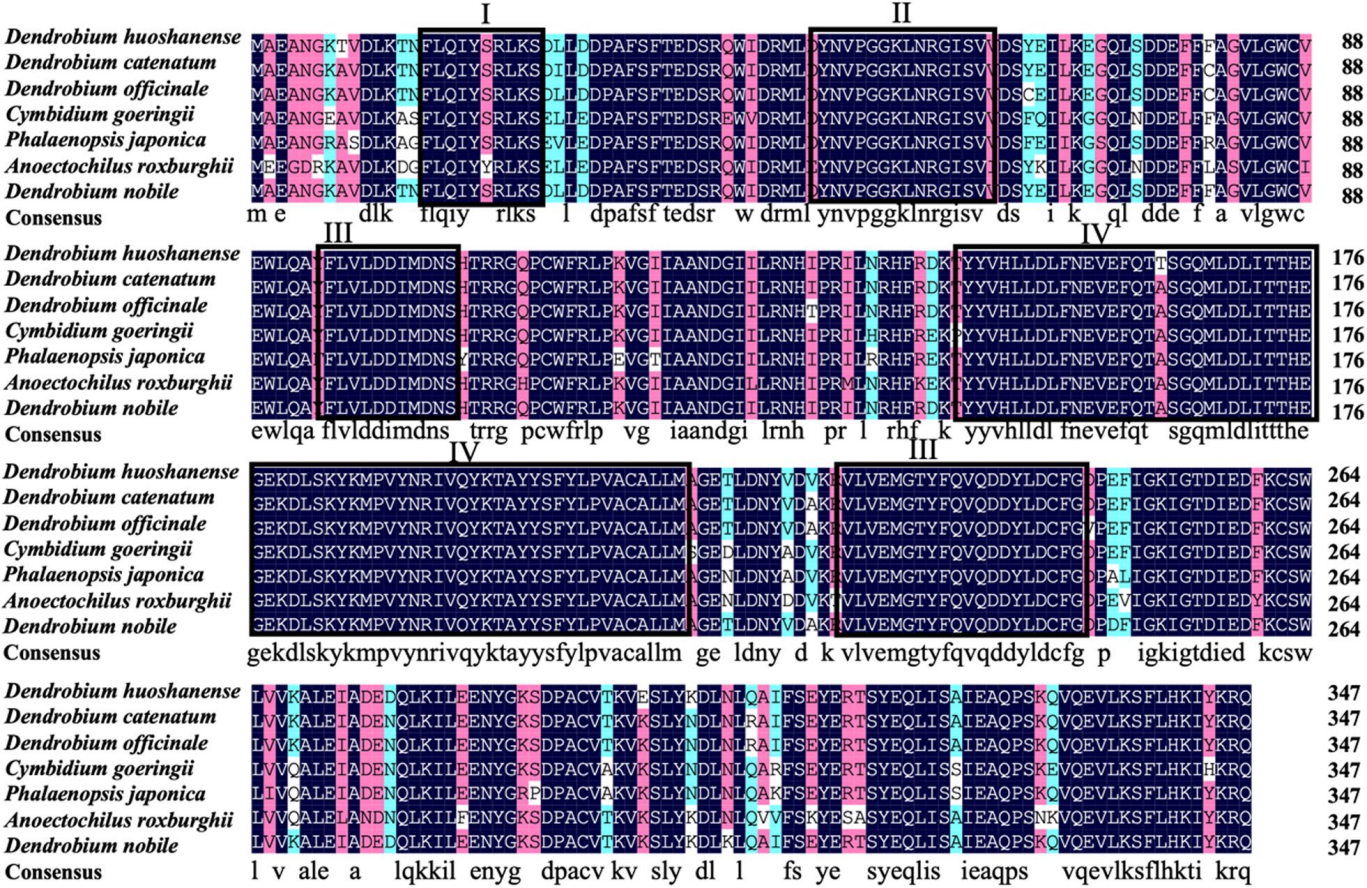
Amino acid sequence alignment of the DnFPPS with FPPS from other plants

**Fig. 3:**
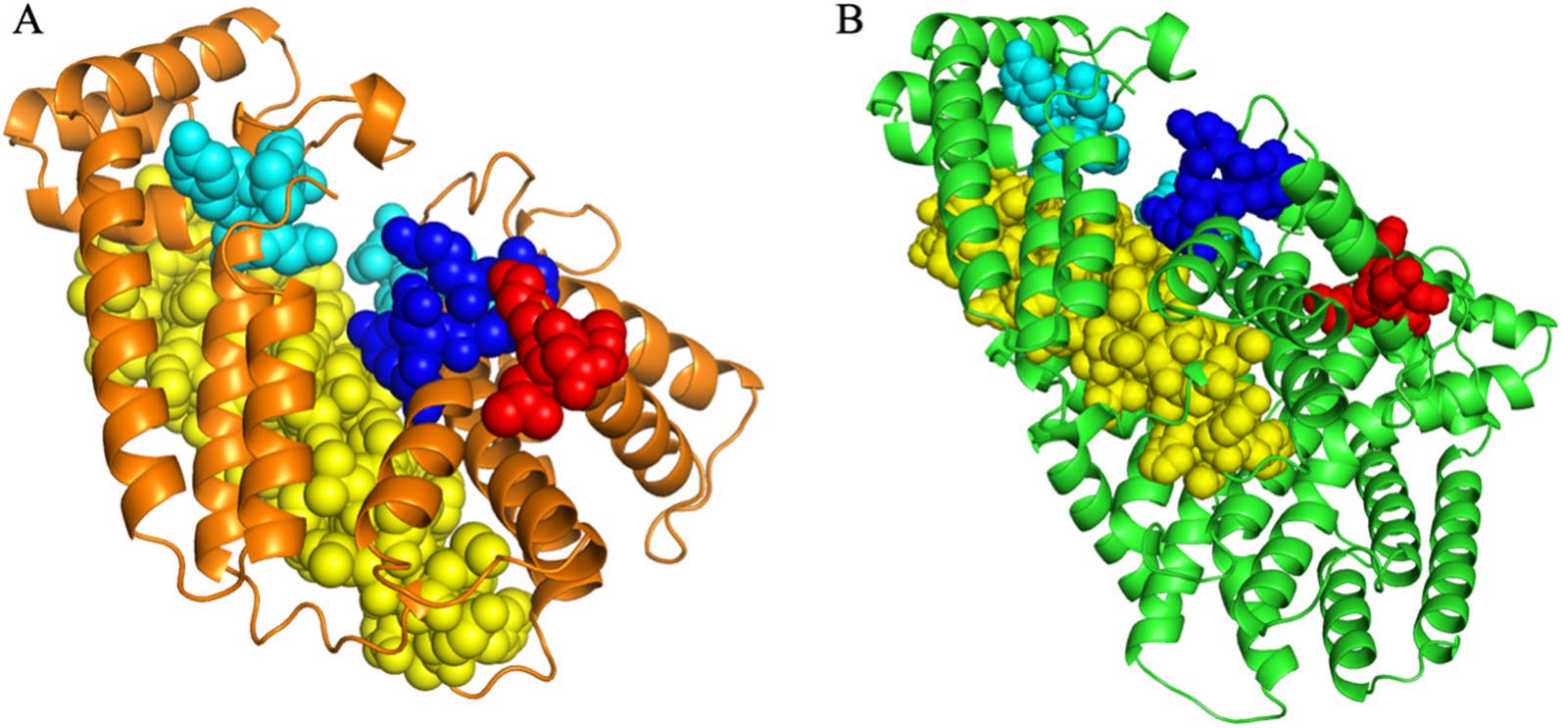
Spatial structure models and domain analysis of FPPS gene. **A** 7bux.1. A model, **B** DnFPPS1 model; sequence identity 73.56%. Conserved regions, motif I, motif II, motif III and motif IV, are separately depicted as spheres in red, blue, cyan and yellow

On line BLASTp result showed that the deduced DnFPPS was in high similarity in amino acid sequence with FPPSs in other plant species, such as DhFPPS from *Dendrobium huoshanense* (98.28%) (accession no. AHC30884.1), DcFPPS from *Dendrobium catenatum* (97.99%) (accession no. XP_020678044.1), DoFPPS from *Dendrobium officinale* (97.41%) (accession no. AFX68799.1), CgFPPS from *Cymbidium goeringii* (91.67%) (accession no. AFP19446.1), PjFPPS from *Phalaenopsis japonica* (90.52%) (accession no. AXQ06578.1), and ArFPPS from *Anoectochilus roxburghii* (87.64%) (accession no. AZP53600.1) (Fig. 2). This suggested that DnFPPS belonged to the FPPS superfamily. Sequence alignment of DnFPPS with FPPSs derived from other plant showed that there were at least four conserved regions, named I to IV (Fig. 2). The N-terminal extension of the FPPSs of these species were enriched in basic, hydroxylated and hydrophobic residues, and had the RXXS tetrapeptide (region I), which was the cleavage motif of mitochondrial targeting peptides. The conserved GGKXXR motif was present in region II. And region II was rich in arginine and lysine, indicating that it may be involved in substrate binding (Blanchard and Karst 1993). The highly conserved aspartate-rich motif (DDXXD) was existed in region III, which were perhaps participated in substrate binding by forming a magnesium salt bridge between the pyrophosphate moiety of the isoprenoid substrate and the carboxyl group of the aspartic acid (Ashby and Edwards 1990) and a very conserved motif (region IV). Subcellular localization predicted that DnFPPS was localized in the cytoplasm and plasma membrane with a probability of 87.6%. The tertiary structure prediction of the protein (Fig. 3) was highly similar to that of *Eucommia ulmoides* FPS1(7bux.1. A).

### Phylogenetic analysis

**Fig. 4:**
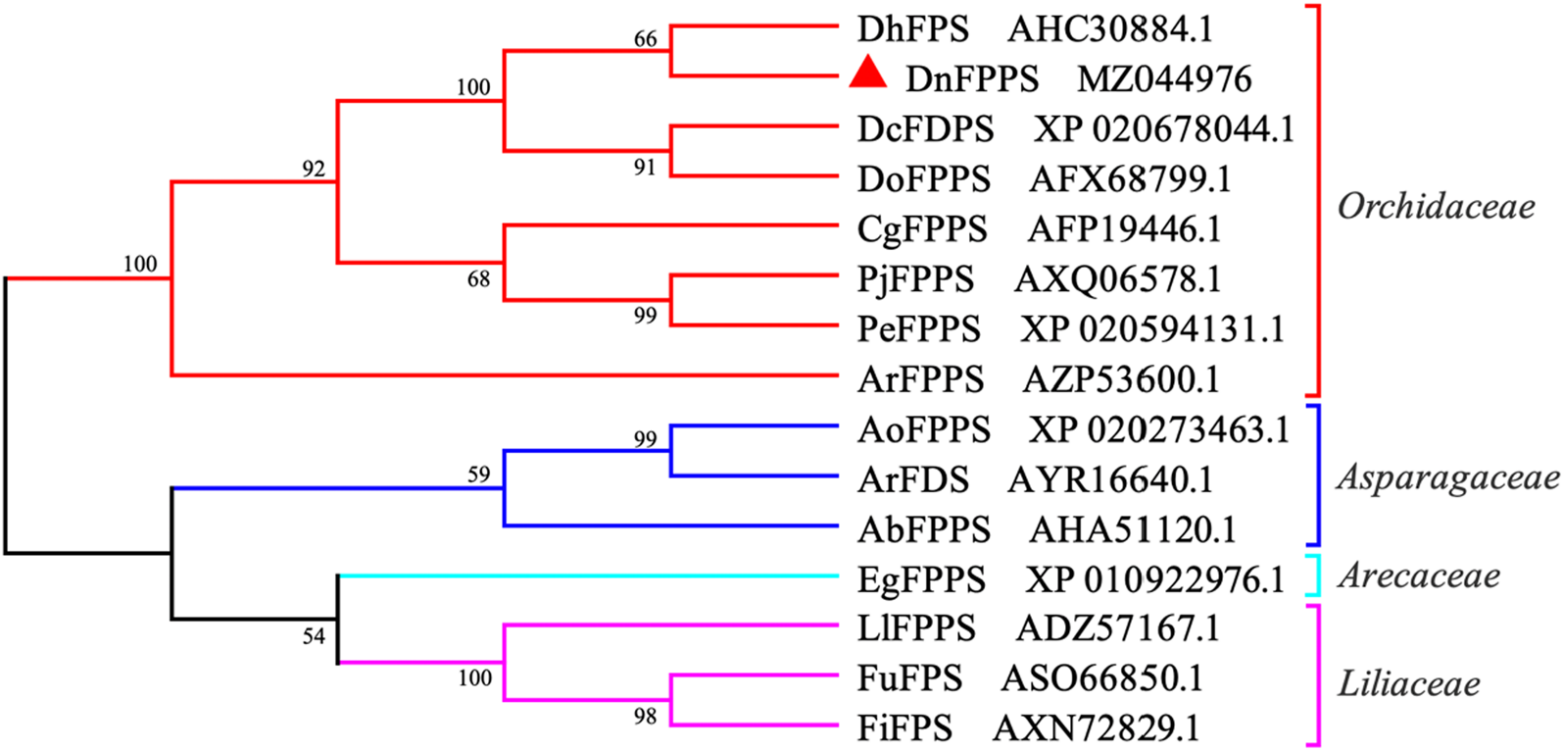
Phylogenetic analysis of amino acid sequences between DnFPPS and other plants FPPS. Maximum Likelihood phylogenetic tree was constructed using MEGA7.0 software. The numbers at nodes represent 1000 bootstrap replicates. The accession numbers were as follows: DhFPS, *Dendrobium huoshanense*; DcFDPS, *Dendrobium catenatum*; DoFPPS, *Dendrobium officinale*; CgFPPS, *Cymbidium goeringii*; PjFPPS, *Phalaenopsis japonica*; PeFPPS, *Phalaenopsis equestris*; ArFPPS, *Anoectochilus roxburghii*; AoFPPS, *Asparagus officinalis*; ArFDS, *Asparagus racemosus*; AbFPPS, *Albuca bracteate*; FuFPS, *Fritillaria unibracteata*; LlFPPS, *Lilium longifloraria*; EgFPPS, *Elaeis guineensis*; FiFPS, *Fritillaria imperialis*

The phylogenetic tree was constructed by 14 plant FPPSs obtained from NCBI. Result showed that the 15 plant FPPSs could be grouped into 4 categories, namely *Orchidaceae*, *Asparagaceae*, *Arecaceae* and *Liliaceae* (Fig. 4). The *Dendrobium* FPPSs were clustered into one subgroup, inluding DnFPPS, DhFPS, DcFDPS and DoFPPS, and the DnFPPS was more colse to DhFPS.

### **Expression and purification of DnFPPS in***E. coli*

**Fig. 5:**
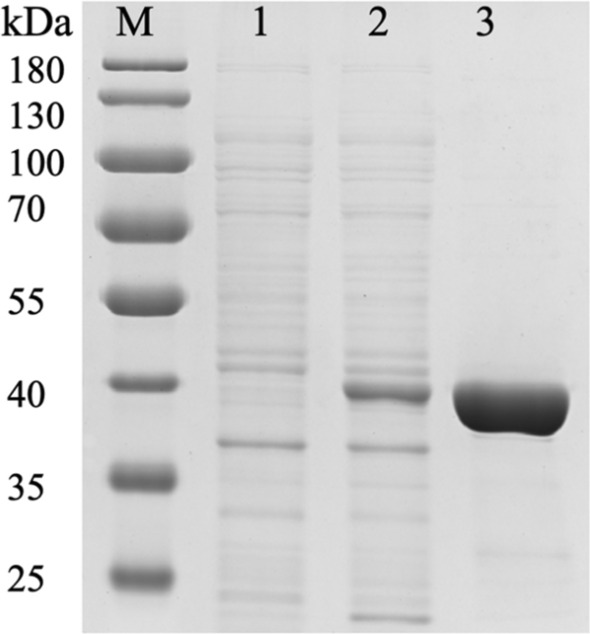
SDS-PAGE analysis of DnFPPS recombinant protein. M: protein standard marker (180 kDa); 1: The culture supernatant (negative control) from mock transformant *E. coli* BL21(DE3) containing expression vector pET-28a; 2: culture supernatant culture supernatant (positive control) from transformant *E. coli* BL21(DE3) containing expression vector pET-28a-DnFPPS; 3: purified DnFPPS protein

In order to screen out clones that can produce the DnFPPS protein, 1 positive clone was inoculated into 25 mL shaking flasks containing 25 mL Ampicillin (100 mg/mL), and cultivated at 37 ℃,150 rpm to OD = 0.8–1.0. Then, 0.5 mM isopropyl-beta-d-thiogalactopyranoside (IPTG) was added to the culture and incubated at 16 °C, 150 rpm overnight. Then the cells were disrupted by ultrasonic cell crushing apparatus. Subsequently, sodium dodecyl sulfate-polyacrylamide gel electrophoresis (SDS-PAGE) was used to detect the expression of the recombinant DnFPPS (Fig. 5). SDS-PAGE was performed on a 10% flowing gel, and the band size was observed after Coomassie brilliant blue staining and decolorization. The results showed that the molecular weight of DnFPPS was approximately 40 kDa (Fig. 5, line 2), which was consistent with the predicted molecular weight. Finally, the DnFPPS was purified by His-tag by Ni^2+^ chelating chromatography, which showed an obvious single band (Fig. 5, line 3). These above results indicated that the DnFPPS protein were successfully expressed, and purified, and could be used for subsequent enzyme activity detection.

### The product of FPPS was determined by gas chromatography–mass spectrometer (GC–MS)

**Fig. 6:**
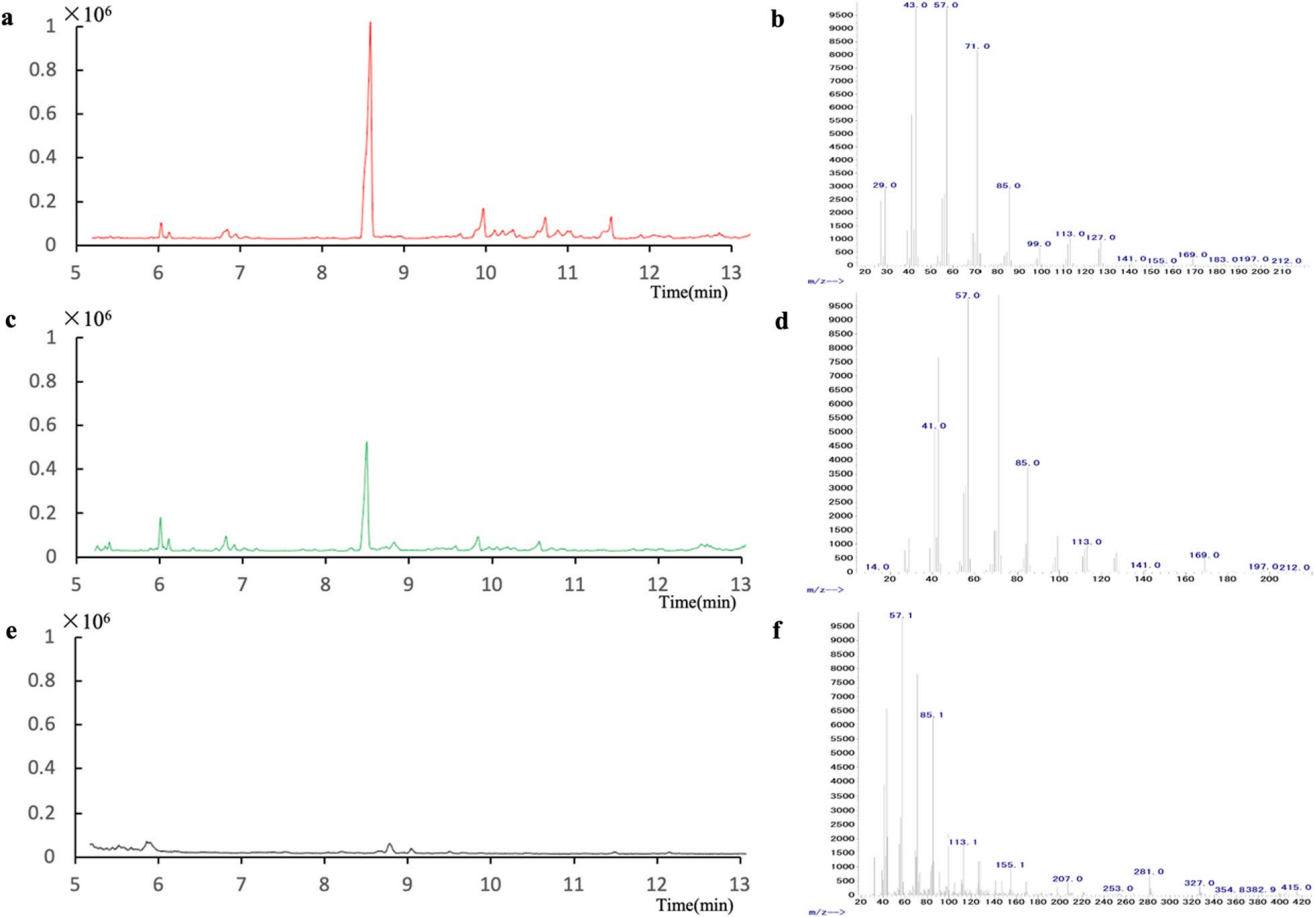
GC-MS was used to detect the catalytic product of *DnFPPS* encoding DnFPPS. **a** FPP-derived farnesane. **b** Mass spectrum of farnesane. **c** Samples extracted from the in vitro reaction mixture containing purified DnFPPS. **d** Mass spectrum of the 8.67-min peak in **c**. **e** A sample extracted from an in vitro reaction mixture containing products from a mock-transformant *E. coli* mock-purification protocol containing the expression vector pET-28a. **f** Mass spectrum of the 8.85-min peak in **e**

In order to explore the function of DnFPPS, the purified DnFPPS and substrates (IPP and DMAPP) were added to the same reaction system. After the reaction was catalyzed overnight at 30 °C, the mixture was extracted with l-butanol saturated with water, and then the diphosphate group was hydrolyzed with potato acid phosphatase at 37 °C. Secondly, the hydrolysate was extracted with n-hexane, filtered with a 0.22 mM filter membrane, and the filtrate was analyzed by GC-MS. As shown in Fig. 6, comparing the retention time of the sample with the retention time of FPP-derived farnesane (Fig. 6a), it was confirmed that DnFPPS could catalyze DMAPP and IPP to generate FPP (Fig. 6c). In contrast, no such product peak was detected in the sample extracted from the in vitro reaction mixture containing the mock-transformed *E. coli* mock-purification protocol containing the expression vector pET-28a (Fig. 6e). These results indicated that *DnFPPS* of *D. nobile* is a functional FPPS.

### Analysis of the expression profiles of dendrobine biosynthetic pathway genes under MeJA treatments

Seven key enzyme genes, including *AACT*, *HMGS*, *HMGR*, *MVD*, *PMK* and *MK* in the upstream of FPPS, and a downstream *TPS* potentially involved in the biosynthesis of dendrobine were screened from the transcriptome data (Li et al. 2017), and their expression levels were verified by qRT-PCR (Additional file 1: Fig. S4). These Results showed that *AACT*, *HMGS*, *HMGR*, *PMK* and *MK* had no significant correlation with *DnFPPS* (Additional file 1: Table S3) and their relative expression levels were shown in Additional file 1: Fig. S4. While, *MVD* and *TPS21* were significantly correlated with *DnFPPS* gene, and the correlation coefficient was 0.638 and 0.631, respectively.

## Discussion

As a class of natural hydrocarbon compounds with isoprene as the structural unit, terpenoids are widely distributed, with diverse in types and rich in structure. FPPS is a key enzyme in the biosynthetic pathway of terpenoids in plants. So far, many scholars have studied the key enzyme genes of *fpps* (Abate et al. 2017; Cao et al. 2012; Cheng and Li 2019; Deng et al. 2016; Dozier and Distefano 2012; Pérez-Castrillón et al. 2014). It is generally believed that FPP is an essential compound for most linear isoprenoids, sterols, and sesquiterpenes in the biosynthesis (Kajiura et al. 2017). Decades of FPP synthesis studies have identified and characterized many *fpps* genes from bacteria, insects, plants and mammals (Kajiura et al. 2017). In *D. nobile*, FPP is believed to be a key compound in the biosynthetic pathway of dendrobine, and thus there is a meaningful study to elucidate the biosynthetic mechanism of FPP and characterize DnFPPS (Gong et al. 2021). For example, FPP is catalyzed by caryophyllene synthase, germacrene D synthase, squalene synthase and amorpha-4, 11-diene synthase to generate caryophyllene, germacrene D, squalene and amorphadiene, respectively (Gong et al. 2021).Therefore, it is particularly important to explore the sesquiterpenoid skeleton intermediates formed by DnFPPS in the biosynthesis pathway of dendrobine.

Due to dendrobine having sesquiterpene skeleton, its biosynthetic pathway is similar to other sesquiterpene compounds. Dendrobine was separated from *D.nobile* in early 1932 by H. Suzuki (Suzuki 1932). Later, in 1935, Chen isolated the compound from *D. nobile* (Chen and Chen 1935). However, its sesquiterpene skeleton structure was not initially identified until 1963 by Y. Inubushi (Inubushi et al. 1963). Dendrobine was identified as a tetracyclic system with a five-membered lactone ring structure, containing N-methyl and oxygen in the form of γ -lactone, and its molecular formula is C_16_H_25_NO_2_ (Sando et al. 2008). Subsequently, the tricyclic skeleton structure of dendrobine was synthesized (Kaneko 1970). The complete biosynthetic pathway of dendrobine was reported, the complex steps and low recovery of dendrobine were identified (Corbella et al. 1975; Lee et al. 2017b; Sha et al. 1997; Yamazaki et al. 1966). Dendrobine is still the main source of *dendrobium* plant extraction and the key enzymes genes in the biosynthetic pathway of dendrobine are rarely reported. Therefore, how to reconstruct dendrobine biosynthesis pathway in vitro has become a key problem to be solved urgently.

In this study, we have isolated *DnFPPS* from *D. nobile*, and analyzed the characterization of DnFPPS by bioingformatics approach. The full length of *DnFPPS* is 1231 bp with an open reading frame of 1047 bp encoding 348 amino acids. It was predicted that the relative molecular mass of DnFPPS protein was 40.31 kDa and the isoelectric point was 5.09. Through homology analysis and phylogenetic tree analysis, we known that the nucleotide sequence similarity of the FPPS between *D. nobile*. and *D. huoshanense* (AHC30884.1) was 98.28%. DnFPPS was expressed in the recombinant *E. coli* and purified. In order to determine *DnFPPS* as a functional gene encoding FPPS, purified DnFPPS was used to catalyze DMAPP and IPP to synthesize FPP. GC-MS test confirmed that the DnFPPS gene from *D. nobile* is a functional gene encoding FPPS. The results indicated that the activity of FPPS in *D. nobile* was nonlinearly correlated with the content of dendrobine. As far as we know, this is the first report about gene cloning and characterization from *D. nobile*. Our results will be helpful to facilitate the investigation of its structure, expression and the role of FPPS in the control of dendrobine biosynthesis in *D. nobile*. In addition, we also explored some other genes involved in dendrobine biosynthesis pathway, and found that the terpenoid synthase gene (*TPS21*) was significantly correlated with *DnFPPS* gene and its relative expression level was consistent with that of *DnFPPS* gene. Therefore, we speculated that *TPS21* might be a key enzyme gene involved in dendrobine biosynthesis pathway. However, we cannot exclude other key enzyme genes from participating in the dendrobine biosynthesis pathway, because on the one hand, these genes were a large gene family, and we only selected one of the genes with significant differences in the transcription set induced by mycorrhizal fungi (MF23) as a reference for relative expression levels. On the other hand, other genes may be the same function after induction of methyl jasmonate, but we haven’t found it yet.

To date, we have initially explored the function of FPPS, which is far from enough for the biosynthetic pathway of dendrobine. For example, knockdown of the MpFPPS1/2 gene significantly reduced the proportion of droplets released by aphids and the (E)-β-farnesene content (Cheng and Li 2019), this result suggest that both *fpps* genes are involved in the production of (E)-β-farnesene in *Myzus persicae*. In addition, more experiments should be conducted in the future to further explore the role and molecular mechanism of FPPS in the biosynthesis of dendrobine. Future research will focus on the use of inhibition, transgene, RNAi and other technologies to verify the function of the *DnFPPS*, in order to intuitively and accurately clarify the molecular mechanism of FPPS. For example, the activity of human FPPS, EuFPPS, and PcFPPS is inhibited by IPP, suggesting that the active site (which contributes to the binding of allyl substrates) is occupied by IPP close to the first DDxxD motif (Kajiura et al. 2017). Previous studies have shown that two IPP molecules bind to avian FPPS monomers, indicating that IPP can bind to donor and allyl sites (Kajiura et al. 2017; King and Rilling 1977). Thus, there may be potential utilization sites near the active sites of FPPS to control dendrobine biosynthesis. Meanwhile, it has been shown that long N-terminal sequences do not affect the function of PcFPPS, because PcFPPS contains a RxxS motif, the cleavage site of mitochondrial targeting sequence Arg67-Ser70 (Frick et al. 2013).In general, FPPS enzymes require Mg^2+^ or Mn^2+^ or Co^2+^as a cofactor for FPP synthesis. Such as Co^2+^ acts as a cofactor in EuFPPS, and PcFPPS (Kajiura et al. 2017). Therefore, the role of cofactors should also be taken into account when reconstructing dendrobine biosynthesis in vitro.

In short, dendrobine is the main active compound of *D. nobile*, which has a variety of biological activities and considerable economic value. However, its biosynthesis is an extremely complex and dynamic process that is regulated by multiple factors. It is still unclear how the activity of its key enzymes during the growth of *D. nobile* is affected by environmental factors, and the relationship between the synthesis of secondary metabolites and key enzymes is unclear. Studies have shown that mycorrhizal fungi can increase the production of dendrobine. However, its molecular mechanism needs to be further explored (Li et al. 2017).In the future, we can focus on studying whether environmental factors in the habitat of *D. nobile* affect the expression of *FPPS*, so as to achieve the goal of increasing the production of dendrobine and provide scientific basis for improving the medicinal quality and economic value of *D. nobile*.

## Supplementary information

**Additional file 1: Table S1**. Primers used in thisstudy. Primer sequences for the restriction sites are shown in bold. **Table S2**.Relevant FPPSs sequences for phylogenetic analysis. **Table S3**. a. Correlationanalysis between AACT and FPPS. b. Correlation analysis between HMGR and FPPS. c.Correlation analysis between HMGS and FPPS. d. Correlation analysis between MK andFPPS. e. Correlation analysis between PMK and FPPS. f. Correlation analysisbetween MVD and FPPS. g. Correlation analysis between TPS21 and FPPS. **Fig.S1** The MVA and MEP pathways are the main steps in the synthesis ofdendrobine. **Fig. S2.** The relative expression of *DnFPPS* atdifferent tissues in *D.nobile. ***Fig. S3** Agarose gelelectrophoresis of the core fragment amplification products of *Dnfpps*. **Fig. S4** The relative expression ofdifferent genes at different time points in *D.nobile* after MeJAtreatment.

## Data Availability

The raw data supporting the conclusions of this manuscript will be made available by the authors, without undue reservation, to any qualified researcher.

## Abbreviations

*D. nobile*: * Dendrobium nobile* Lindl
*E. coli*: BL21(DE3) *Escherichia coli* BL21(DE3)
IPP: Isopentenyl diphosphate
DMAPP: Dimethylallyl diphosphate
FPP: Farnesyl diphosphate
MeJA: Methyl Jasmonate
ORF: Open reading frame
IPTG: Isopropyl-beta-d-thiogalactopyranoside
GC-MS: Gas chromatography-mass spectrometer
SDS-PAGE: Sodium dodecyl sulfate-polyacrylamide gel electrophoresis
