# Supplementary material for: Functional characterization of a farnesyl diphosphate synthase from Dendrobium nobile Lindl
Source: AMB Express. 2022 Oct 6;12:129. doi: 10.1186/s13568-022-01470-2 (PMC9537409; doi:10.1186/s13568-022-01470-2)
Supplement: Supplementary file 1 — Additional file 1: Table S1. Primers used in thisstudy. Primer sequences for the restriction sites are shown in bold. Table S2.Relevant FPPSs sequences for phylogenetic analysis. Table S3. a. Correlationanalysis between AACT and FPPS. b. Correlation analysis between HMGR and FPPS. c.Correlation analysis between HMGS and FPPS. d. Correlation analysis between MK andFPPS. e. Correlation analysis between PMK and FPPS. f. Correlation analysisbetween MVD and FPPS. g. Correlation analysis between TPS21 and FPPS. Fig.S1 The MVA and MEP pathways are the main steps in the synthesis ofdendrobine. Fig. S2. The relative expression of DnFPPS atdifferent tissues in D.nobile. Fig. S3 Agarose gelelectrophoresis of the core fragment amplification products of Dnfpps. Fig. S4 The relative expression ofdifferent genes at different time points in D.nobile after MeJAtreatment. [file 13568_2022_1470_MOESM1_ESM.docx]

**Table S1. Primers used in this study. Primer sequences for the restriction sites are shown in bold.**

| **Primer name** | **Plasmid** | **Forward/ Reverse** | **Sequence** |
| --- | --- | --- | --- |
| FPPS-1-F | pMD19-T | Forward | ATGGCGGAAGCGAACG |
| FPPS-1-R | pMD19-T | Reverse | TTAGTTGTTCGGATTCATTTTGG |
| FPPS-1-3-RACE-F | pMD19-T | Forward | AGCTGAAGGCTGGATCATCG |
| FPPS-1-5-RACE-R | pMD19-T | Reverse | AGGCGGTGGATCTGAAAACC |
| FPPS-1-short-RACE | pMD19-T | Forward/ Reverse | CTAATACGACTCACTATAGGGC |
| FPPS-1-ORF-F | pMD19-T | Forward | ATGGCGGAAGCGAACGG |
| FPPS-1-ORF-R | pMD19-T | Reverse | CTACTTCTGTCTTTTGTAGATC |
| FPPS-1-F-BamHI | pET-28a | Forward | CG**GGATCC**ATGGCGGAAGCGAACGG |
| FPPS-1-R-xhoI | pET-28a | Reverse | CCG**CTCGAG**CTACTTCTGTCTTTTGTAGATC |
| FPPS-1-RT-F | N/A | Forward | TGTACCAGGAGGGAAGCTCA |
| FPPS-1-RT-R | N/A | Reverse | TAAACCAGCAAGGCTGACCA |
| GAPDH-F | N/A | Forward | TAAGGCTGCTATAAAGGAAGAATC |
| GAPDH-R | N/A | Reverse | GACCTGCTGTCACCCAAGAA |
| AACT-F | N/A | Forward | AGCCTTCCTTGGTTCATTGTCATCT |
| AACT-R | N/A | Reverse | CTGTGCTGCTATCATTGTTGCCTTC |
| HMGS-F | N/A | Forward | GCGTTCAGCAGGAATCATTAGAGG |
| HMGS-R | N/A | Reverse | TCTCAAGCAAGGAAGTGACAACTGT |
| HMGR-F | N/A | Forward | GCCAGCAACATAGTATCAGCCATCT |
| HMGR-R | N/A | Reverse | CACCACCAACAGTTCCTACCTCAAT |
| MVD-F | N/A | Forward | GGCTACGACGACTGTTGCTGTTAG |
| MVD-R | N/A | Reverse | CTGAATGCCTCGCTTCTCATCCTC |
| PMK-F | N/A | Forward | TGGAGGAAGCTATATGCCTGATGTG |
| PMK-R | N/A | Reverse | CTGCCAGTCGTGTCCGTGTT |
| MK-F | N/A | Forward | GCCTGCTCAATTCCTGCTATTAACA |
| MK-R | N/A | Reverse | GCGAGTTCTGACCTCCATTATTCCT |
| TPS21-F | N/A | Forward | GTTGCGGCATCGGAAGGTCATTA |
| TPS21-R | N/A | Reverse | AGGGCGAGATGGAGTGATGGAAA |
| N/A=not available |  |  |  |

**Table S2. Relevant FPPSs sequences for phylogenetic analysis.**

| Enzymes | Gene name | Species | GenBank accession number |
| --- | --- | --- | --- |
| farnesyl pyrophosphate synthetase 1 | *DhFPS* | Dendrobium huoshanense | AHC30884.1 |
|  | *DcFDPS* | Dendrobium catenatum | XP_020678044.1 |
|  | *DoFPPS* | Dendrobium officinale | AFX68799.1 |
|  | *CgFPPS* | Cymbidium goeringii | AFP19446.1 |
|  | *PjFPPS* | Phalaenopsis japonica | AXQ06578.1 |
|  | *PeFPPS* | Phalaenopsis equestris | XP_020594131.1 |
|  | *ArFPPS* | Anoectochilus roxburghii | AZP53600.1 |
|  | *AoFPPS* | Asparagus officinalis | XP_020273463.1 |
|  | *ArFDS* | Asparagus racemosus | AYR16640.1 |
|  | *AbFPPS* | Albuca bracteata | AHA51120.1 |
|  | *FuFPS* | Fritillaria unibracteata | ASO66850.1 |
|  | *LlFPPS* | Lilium longifloraria | ADZ57167.1 |
|  | *EgFPPS* | Elaeis guineensis | XP_010922976.1 |
|  | *FiFPS* | Fritillaria imperialis | AXN72829.1 |

**Table S3. a. Correlation analysis between AACT and FPPS.**

|  |  | AACT | FPPS |
| --- | --- | --- | --- |
| AACT | Pearson Correlation | 1 | .183 |
|  | Sig. (2-tailed) |  | .570 |
|  | N | 12 | 12 |
| FPPS | Pearson Correlation | .183 | 1 |
|  | Sig. (2-tailed) | .570 |  |
|  | N | 12 | 12 |

**Table S3. b. Correlation analysis between HMGR and FPPS.**

|  |  | HMGR | FPPS |
| --- | --- | --- | --- |
| HMGR | Pearson Correlation | 1 | .325 |
|  | Sig. (2-tailed) |  | .302 |
|  | N | 12 | 12 |
| FPPS | Pearson Correlation | .325 | 1 |
|  | Sig. (2-tailed) | .302 |  |
|  | N | 12 | 12 |

**Table S3. c. Correlation analysis between HMGS and FPPS.**

|  |  | HMGS | FPPS |
| --- | --- | --- | --- |
| HMGS | Pearson Correlation | 1 | .183 |
|  | Sig. (2-tailed) |  | .570 |
|  | N | 12 | 12 |
| FPPS | Pearson Correlation | .183 | 1 |
|  | Sig. (2-tailed) | .570 |  |
|  | N | 12 | 12 |

**Table S3. d. Correlation analysis between MK and FPPS.**

|  |  | MK | FPPS |
| --- | --- | --- | --- |
| MK | Pearson Correlation | 1 | -.051 |
|  | Sig. (2-tailed) |  | .875 |
|  | N | 12 | 12 |
| FPPS | Pearson Correlation | -.051 | 1 |
|  | Sig. (2-tailed) | .875 |  |
|  | N | 12 | 12 |

**Table S3. e. Correlation analysis between PMK and FPPS.**

|  |  | PMK | FPPS |
| --- | --- | --- | --- |
| PMK | Pearson Correlation | 1 | .423 |
|  | Sig. (2-tailed) |  | .170 |
|  | N | 12 | 12 |
| FPPS | Pearson Correlation | .423 | 1 |
|  | Sig. (2-tailed) | .170 |  |
|  | N | 12 | 12 |

**Table S3. f. Correlation analysis between MVD and FPPS.**

|  |  | MVD | FPPS |
| --- | --- | --- | --- |
| MVD | Pearson Correlation | 1 | .638* |
|  | Sig. (2-tailed) |  | .025 |
|  | N | 12 | 12 |
| FPPS | Pearson Correlation | .638* | 1 |
|  | Sig. (2-tailed) | .025 |  |
|  | N | 12 | 12 |

**Table S3. g. Correlation analysis between TPS21 and FPPS.**

|  |  | TPS21 | FPPS |
| --- | --- | --- | --- |
| TPS21 | Pearson Correlation | 1 | .631* |
|  | Sig. (2-tailed) |  | .028 |
|  | N | 12 | 12 |
| FPPS | Pearson Correlation | .631* | 1 |
|  | Sig. (2-tailed) | .028 |  |
|  | N | 12 | 12 |

* At level 0.05 (two-tailed), the correlation was significant.


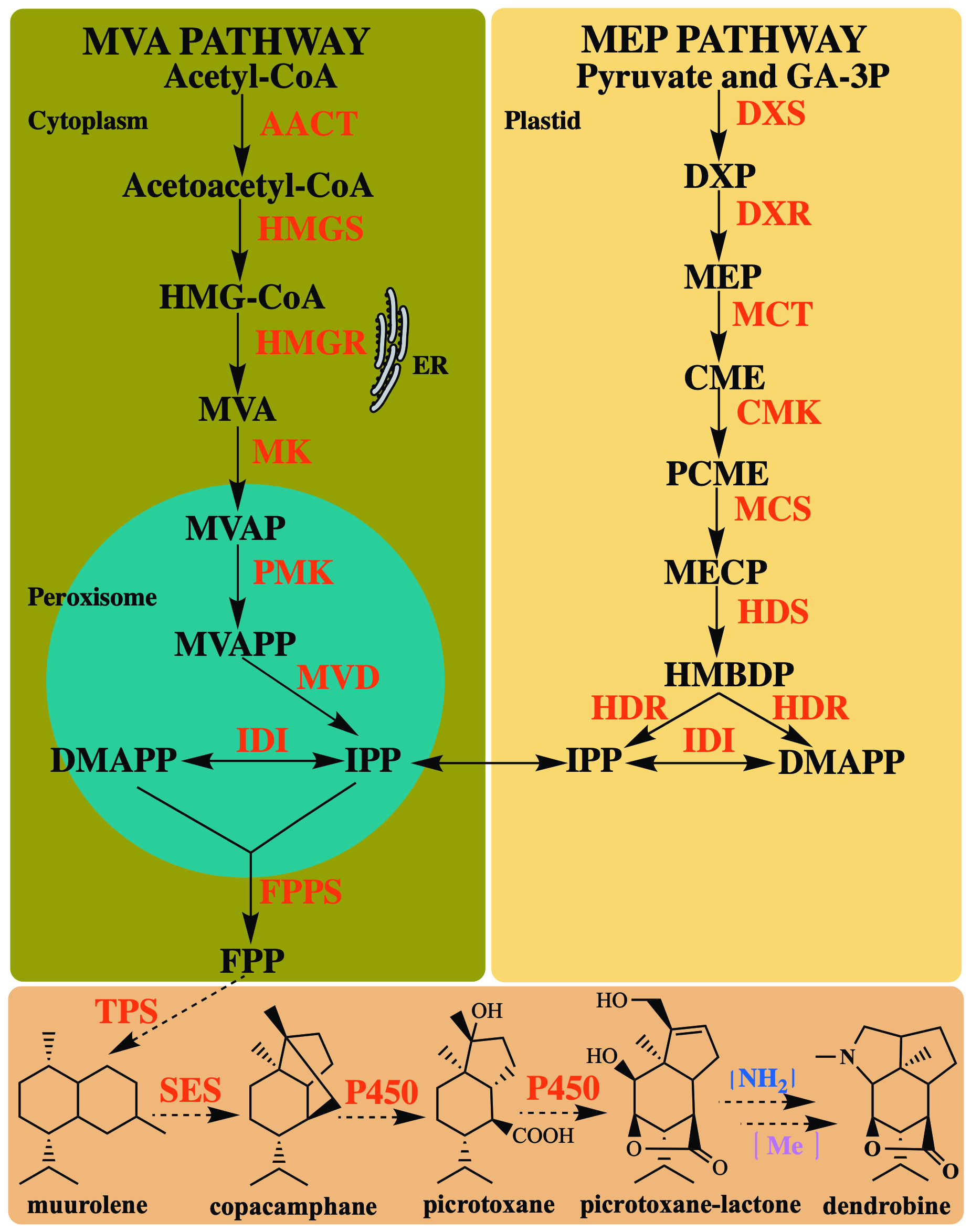


**Fig. S1 The MVA and MEP pathways are the main steps in the synthesis of dendrobine.**

The abbreviations used are: AACT, acetoacetyl-CoA thiolase; HMGS, 3-hydroxy-3-methylglutaryl- CoA synthase; HMGR, 3-hydroxy- 3-methylglutaryl-CoA reductase; MK, mevalonate kinase; MVAP, mevalonate 5-phosphate; PMK, phosphomevalonate kinase; MVAPP, mevalonate diphosphate; MVD, mevalonate diphosphate decarboxylase; IPP, isopentenyl diphosphate; DMAPP, dimethylallyl diphosphate; IDI, isopentenyl diphosphate isomerase; FPPS, farnesyl diphosphate synthase; FPP, farnesyl diphosphate; GA-3P, D-glyceraldehyde 3-phosphate; DXS, 1-deoxy-D-xylulose 5-phosphate synthase; DXR, 1-deoxy-D-xylulose 5-phosphate reductoisomerase; MCT, 2-C-methyl-D-erythritol 4-phosphate cytidylyltransferase; CMK, 4-(cytidine 5ʹ-diphospho)-2-C-methyl-D-erythritol kinase; MDS, 2-C-methyl-D-erythritol 2,4-cyclodiphosphate synthase; HDS, (E)-4- hydroxy-3-methylbut-2-enyl diphosphate synthase; HDR, (E)-4-hydroxy-3-methylbut-2-enyl diphosphate reductase; TPS, terpenoids synthase; SES, sesquiterpenoid synthase; P450, cytochrome P450 oxidase.


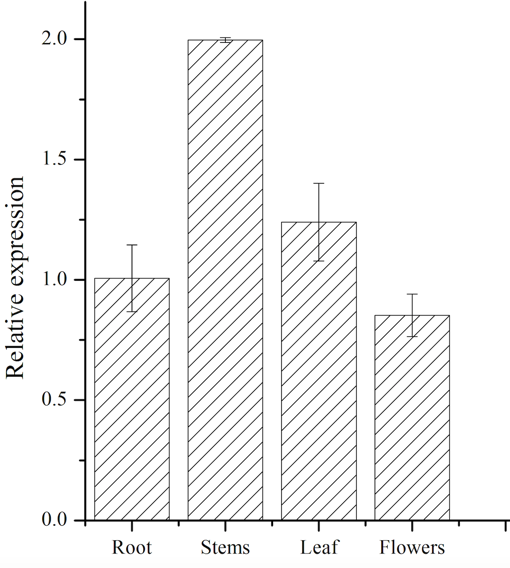


**Fig. S2 The relative expression of *DnFPPS* at different tissues in *D.nobile***


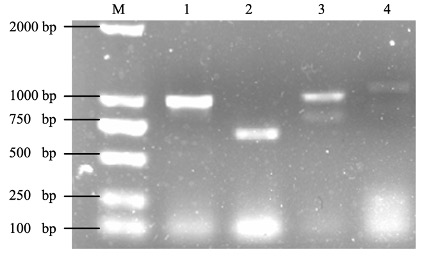


**Fig. S3 Agarose gel electrophoresis of the core fragment amplification products of *Dnfpps***

Note: M: DL2000 Marker; 1: ORF product; 2: 5′-UTR product; 3: 3′-UTR product; 4: Full-length gene product.


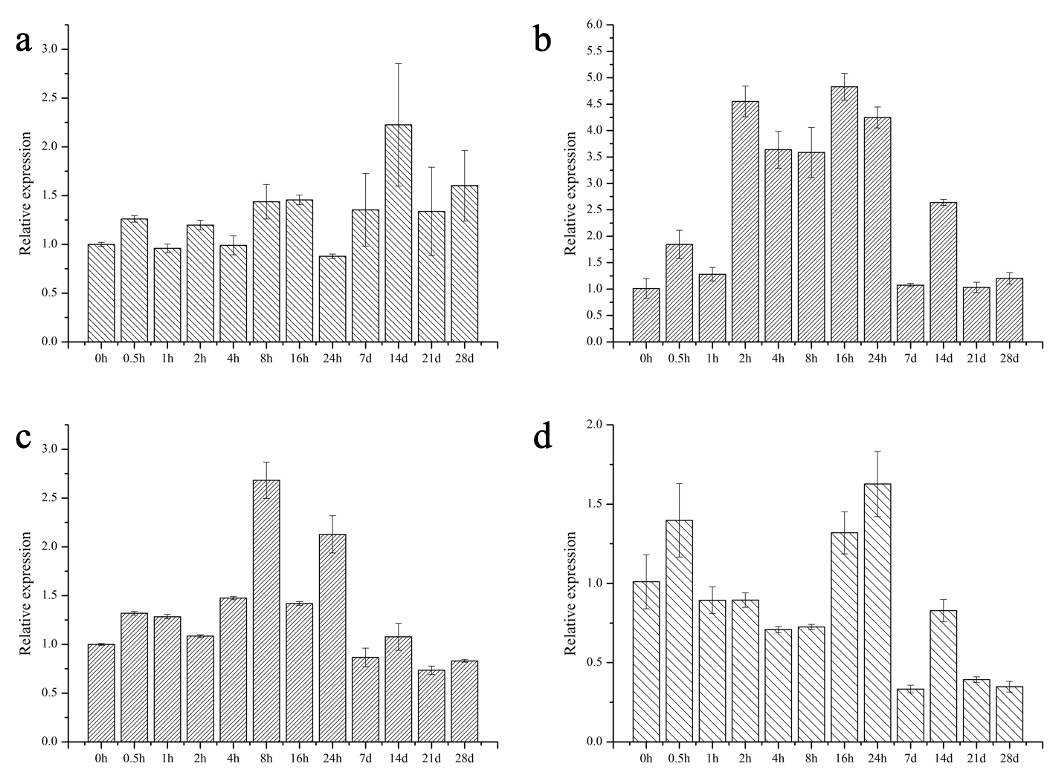


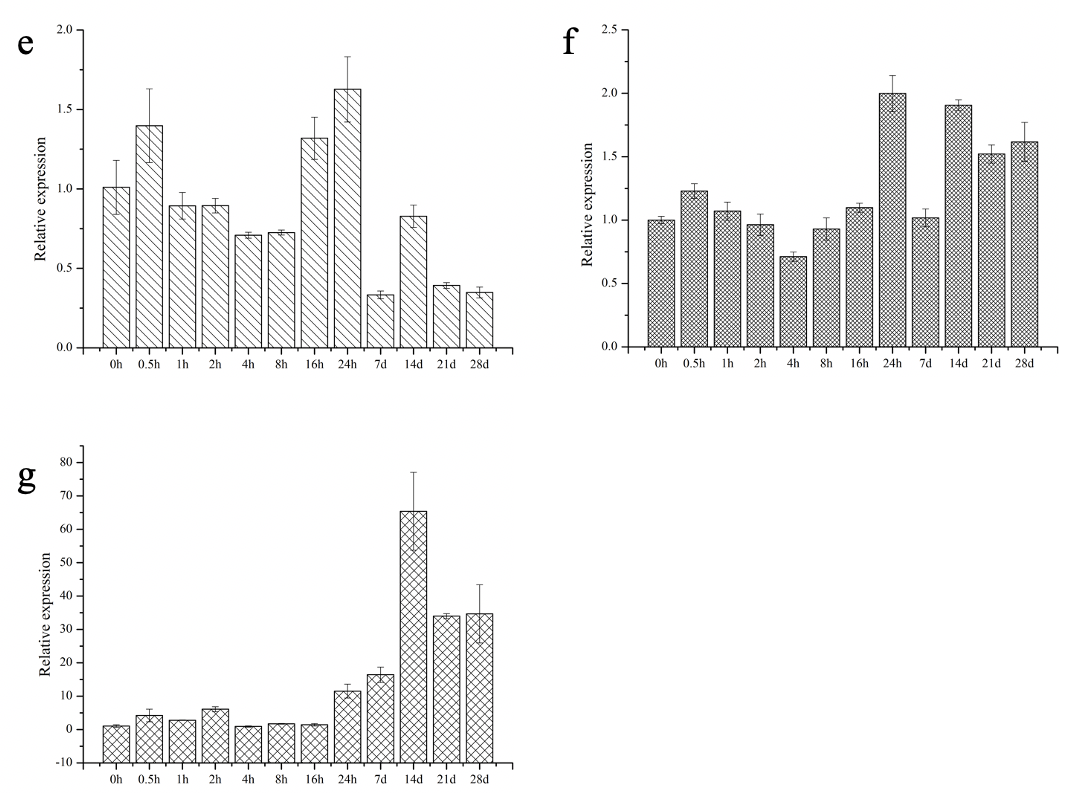


**Fig. S4 The relative expression of different genes at different time points in *D.nobile* after MeJA treatment.** The a, b, c, d, e, f and g represented *AACT*, *HMGR*, *HMGS*, *PMK*, *MK*, *MVD* and *TPS21*, respectively.
